# Ursodeoxycholic Acid Protects Against Arsenic Induced Hepatotoxicity by the Nrf2 Signaling Pathway

**DOI:** 10.3389/fphar.2020.594496

**Published:** 2020-10-16

**Authors:** Chao Li, Sheng Zhang, Liming Li, Qing Hu, Shen Ji

**Affiliations:** ^1^ School of Pharmacy, Shanghai University of Traditional Chinese Medicine, Shanghai, China; ^2^ NMPA Key Laboratory for Quality Control of Traditional Chinese Medicine, Shanghai Institute for Food and Drug Control, Shanghai, China; ^3^ School of Pharmacy, Tianjin University of Traditional Chinese Medicine, Tianjin, China

**Keywords:** arsenic, ursodeoxycholic acid, hepatocytes, nuclear factor e2-related factor (nrf2), oxidative stress, apoptosis

## Abstract

Arsenic is ubiquitous toxic metalloid responsible for many human diseases all over the world. Contrastingly, Ursodeoxycholic acid (UDCA) has been suggested as efficient antioxidant in various liver diseases. However, there are no reports of the effects of UDCA on arsenious acid [As(III)]-induced hepatotoxicity. The objective of this study is to elucidate the protective actions of UDCA on As(III)-induced hepatotoxicity and explore its controlling role in biomolecular mechanisms *in vivo* and *in vitro*. The remarkable liver damage induced by As(III) was ameliorated by treatment with UDCA, as reflected by reduced histopathological changes of liver and elevation of serum AST, ALT levels. UDCA play a critical role in stabilization of cellular membrane potential, inhibition of apoptosis and LDH leakage in LO2 cells. Meanwhile, the activities of SOD, CAT and GSH-Px and the level of TSH, GSH were enhanced with UDCA administration, while the accumulations of intracellular ROS, MDA and rate of GSSG/GSH were decreased *in vivo* and *in vitro*. Further study disclosed that UDCA significantly inhibited As(III)-induced apoptosis through increasing the expression of Bcl-2 and decreasing the expression of Bax, p53, Cyt C, Cleaved caspase-3 and 9. Moreover, UDCA promoted the expression of nuclear Nrf2, HO-1, and NQO1, although arsenic regulated nuclear translocation of Nrf2 positively. When Nrf2 was silenced, the protective effect of UDCA was abolished. Collectively, the results of this study showed that UDCA protects hepatocytes antagonize As(III)-induced cytotoxicity, and its mechanism may be related to activation of Nrf2 signaling.

**Graphical Abstract:**
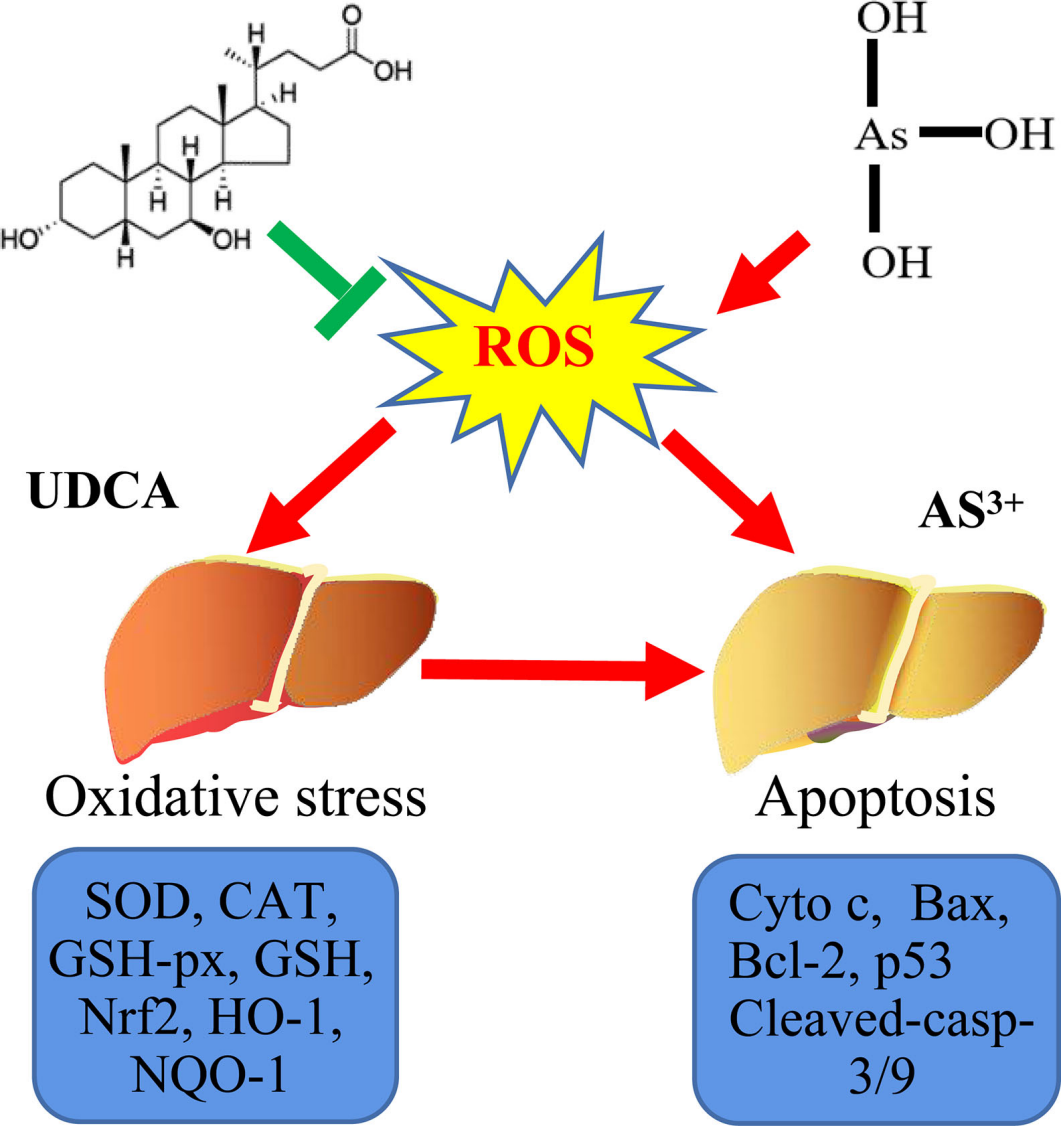
Probable protective mechanism of UDCA against As(III)-mediated cytotoxicity *in vivo* and vitro.

## Introduction

Arsenic compounds are common toxic materials which exposure is associated with numerous disease regime including diabetes mellitus, neurological disorders, and various forms of cancer cardiovascular and peripheral vascular diseases, (Flora, 2011; Sharma et al., 2017; Okereafor et al., 2020). In ancient times, the arsenic compounds were used to treat diseases. Some traditional Chinese medicines were found to contain high arsenic and have been used for more than 3000 years (Huo et al., 2020; Wu et al., 2020). In addition, arsenic induced cells apoptosis is not only the main mechanism of anti-tumor effect, but also one of the main causes of cytotoxic (Wu et al., 2018; Nithyananthan and Thirunavukkarasu, 2020). Therefore, it is of great significance to clarify the damage mechanism of arsenic poisoning and find out the possible regulatory targets (**Graphical Abstract**).

Among the mechanisms of arsenic induced hepatocyte injury, especially oxidative stress mediated hepatocyte apoptosis that is considered as the core event (Nithyananthan et al., 2020). Arsenious acid [As(III)] is believed mainly responsible for the induction of epidemiological toxicity by the production of reactive oxygen species (ROS) and it also exerts genotoxicity (Jiang et al., 2013). In recent years, the study has clearly demonstrated that As(III) administration created redox imbalance tended to oxidation in liver by up-regulating the levels of malondialdehyde (MDA) and H_2_O_2_, thus lead to a down-regulation in the activity of antioxidant enzyme, including glutathione peroxidase (GSH-Px) and catalase (CAT) (Masuda et al., 2018; Tian et al., 2020). In addition, the mammal body has the ability to afford cells protection against oxidative damage by endogenous antioxidant enzymes, such as heme oxygenase-1 (HO-1), NADPH quinone oxidoreductase 1 (NQO1), superoxide dismutase (SOD) (Jiang et al., 2013; Chen et al., 2019; Wang et al., 2019). Nuclear factor erythroid-2-related factor 2 (Nrf2) pathway is one of the most important primary transcription factor that regulates antioxidant enzyme expression. Nrf2 plays an imperative role in cellular defense against oxidative stress, apoptosis, or promotes cell survival by activating antioxidant cascades (Zhang et al., 2013; Tu et al., 2019). Therefore, the reduction of oxidative stress may be a potential mechanism for the treatment of arsenic induced liver injury and other relevant diseases by promoting the activation of Nrf2 pathway.

Ursodeoxycholic acid (UDCA) is a naturally occurring hydrophilic tertiary dihydroxy bile salt used with considerable success in the treatment of liver dysfunction related to various diseases, including primary biliary cirrhosis and chronic active hepatitis (Luketic and Sanyal, 1994). UDCA exhibits a wide range of biological activities, including antioxidant, anti-inflammatory, immunomodulating and anti-apoptotic effects (Makino and Tanaka, 1998; Angulo, 2002), suggesting that UDCA might exert protective effects on hepatocytes. Although the underlying mechanisms of its hepatoprotective effects remain unclear, it is proposed that UDCA can induce GSH synthesis and prevent ROS-induced oxidative damage in hepatocytes through an activation of the Nrf2 pathway (Okada et al., 2008; Arisawa et al., 2009). Herein, our study was conducted to explore whether there is a potential mechanism for oxidative stress in As(III)-induced liver toxicity and also to investigate the putative protective role of UDCA in this scenario.

## Methods and Materials

### Chemicals and Reagents

Arsenious acid solution [As(III), arsenic trioxide was dissolved in alkaline solution and diluted with distilled water to prepare arsenic solutions] and Ursodeoxycholic acid (UDCA) (purity >98%) was obtained from the National Institute of Drugs and Biological Products Control (Beijing, China). Unless otherwise specified, all other analytical grade chemical reagents were purchased from Sigma Chemical Co. (St. Louis, MO, USA). DMEM culture medium, fetal bovine serum (FBS) and antibiotic solution were purchased from Gibco (Grand Island, NY).

### Animals and Treatments

Forty male ICR mice (5-6 weeks) weighing about 18-20 g were provided by Shanghai Lingchang biotechnology Co. LTD. Mice were maintained at a constant temperature (24 ± 2° C) and 40%–60% relative humidity in a 12-h light/dark cycle. They were fed freely water and diet. After a week of adaption, mice were randomly divided into four groups (n = 10) for the treatment of 5% CMCNa (Control group), As(III) group (5 mg/kg), As(III)+UDCA group (30 mg/kg), and UDCA alone group *via* intragastric administration, respectively. The doses of As(III) (Li et al., 2017; Zuo et al., 2017) and UDCA (Mroz et al., 2018; Ouyang et al., 2018; Zhang et al., 2018) were referred to the basis of previously published literature. Mice in the Control groups were administered equal volumes of 5% CMCNa. The body weight was measured once a day. After 7 days, mice blood samples and liver tissues were harvested for further biochemical analysis and histological evaluations. The animal experiment protocol was passed by the Institutional Animal Care and Use Committee of Shanghai Institute for Food and Drug Control (No. SYXK2018-0003). The experiment was carried out in strict accordance with international standards and national regulations on animal care and use. The doses used in this study were the same as those used in previous studies.

### Serum Biochemistry

All mice fasted overnight. The blood samples were collected by removing eye bowl and plasma samples were obtained at 3000 rpm for 10 min for obtaining the plasma samples. Plasma was used for the measurement of blood urea alanine aminotransferase (ALT) and aspartate aminotransferase (AST) by an automatic biochemical analyzer (AU5400; Olympus, Tokyo, Japan).

### Histological Examination

The liver was quickly removed, rinsed briefly in normal saline and then weighed to obtain the wet weight. Then, the liver tissues samples were fixed at 4% paraformaldehyde solution or frozen in liquid nitrogen. Sections of 5 μm thickness were cut and stained with hematoxylin & eosin (HE). The degree of liver lesions was observed under 100× and 400× times magnification optical microscope (Nikon Eclipse TE2000-U, NIKON, Japan).

### Cell Culture, Treatment and Transfection

The human hepatocyte LO2 cell line was purchased from the Shanghai Cell Bank of the Chinese Academy of Sciences. Cells were maintained in DMEM medium supplemented with 10% FBS, 100 U/ml penicillin, and 100 U/ml streptomycin at 37°C and 5% CO2 in a humidified incubator. Cultured LO2 cells were seeded in 96-well plates (200 μl per well) at 5×10^3^cells/ml, or in 6-well plates (2 ml per well) at 5×10^5^ cells/ml and pre-incubated at 37°C in 5% CO2 for 24 h. LO2 cells were incubated with different concentrations of As(III) (0.05, 0.1, 0.2, 0.5, 1, 2, 5, 10 and 20 ppm) were added, separately incubated for 6, 12, 24, 48, 72 h followed. In order to evaluate whether UDCA protected LO2 cells against oxidative damage induced by exposure to As(III), LO2 cells were exposed to 1 ppm As(III) in the presence or absence of pretreatment with UDCA (0, 5, 10, 20, 40, 80, 160 μM) for 24 h. After treatment, cell proliferation and cytotoxicity were assessed using Cell Counting Kit-8 (CCK-8) (Ck04, Dojindo, Japan), 10 µl CCK-8 was added to each well and incubated at 37°C for 1–2 h, the absorbance rate at a wavelength of 450 nm were measured using a microplate reader. The absorbance rate at 450 nm were measured by SpectraMax 250 Microplate Reader (MD, USA). All experiments were repeated six times in triplicate.

To silence Nrf2 expression, aspecific siRNA of Nrf2 and control siRNA (si-NC) were designed and synthesized by Shanghai Genepharma (Shanghai, China). LO2 cells were transfected with 40 nM Nrf2 siRNA and NC siRNA using Lipofectamine 3000 (L3000008, Invitrogen, Carlsbad, USA). After 48 h, knockdown efficiency was analyzed by western blot. After priming with As(III) and UDCA stimulation.

### Lactate Dehydrogenase Cytotoxicity Assay

When the plasma membrane was damaged, lactate dehydrogenase (LDH) was rapidly released into the culture supernatant. The LDH activity in the culture supernatant can be detected to reflect the cell damage. Cultured LO2 cells were seeded in 96-well plates at 5×10^3^ cells/ml pre-incubated at 37 °C in 5% CO2 for 24 h. Cells were exposed to 1 ppm As(III) in the presence or absence of pretreatment with UDCA (0, 5, 10, 20, 40, 80, 160 μM). After the cells were treated, the culture supernatant was collected and analyzed with the LDH cytotoxicity assay kit (C0016, Beyotime, shanghai, China) according to the manufacturer’s protocol and absorbance at 490 nm was measured with a microplate reader.

### Measurement of Intracellular SOD, GSH-Px, CAT, MDA, TSH, GSH, and GSSG Levels

After the mice or cells were treated, cells or liver tissues were lysed in ice-cold RIPA Lysis buffer (P0013B, Beyotime) containing 1 mM phenylmethylsulfonyl fluoride (PMSF). The lysate was collected and put into EP tube, and the protein concentration was detected by BCA reagent method (P0010, Beyotime). Total sulfhydryl groups (TSH) in the cells or liver tissues was analyzed with the Micro Total Mercapto Assay Kit (BC1375, Solarbio, Beijing, China) according to the manufacturer’s protocol. The levels of MDA (SO0131, Beyotime), GSH/GSSG (S0053, Beyotime), and activities of SOD (S0101, Beyotime), GSH-Px (S0058, Beyotime), CAT (S0051, Beyotime) were measured with commercial assay kits according to the manufacturers’ protocols.

### Intracellular ROS Quantification

The fluorescence intensity of intracellular ROS was measured by fluorescent probe DCFH-DA and DHE (S003M and S0063, Beyotime). Afterwards, the treated cells and mice liver were labeled with DCFH-DA (20 mM) for 30 min, and DHE for another 30 min, washed twice with PBS, and then, Liver tissues were embedded in OCT medium and frozen. Sections 5 μM in thickness were prepared, DAPI (100 ng/ml) was used for nuclear counter staining. The fluorescence intensity of cells or tissue sections was observed under a fluorescence microscope (magnification, ×100) (Nikon 80i, Japan).

### Immunofluorescence Assay

Liver tissues were embedded in OCT medium and frozen. Sections 5 μM in thickness were prepared. LO2 cells or tissue sections were fixed for 15 min in 4% paraformaldehyde in PBS. The fixed cells were permeabilized for 20 min with 0.5% Triton-X100 in PBS and then blocked in PBS with 1% goat serum for 30 min. The cells were incubated with the appropriate primary rabbit Bcl2 (ab185828, abcam), Bax (ab32503, abcam), Nrf2 (16396-1-AP, Wuhan, China) and then stained with Alexa Fluor 488-labeled goat anti-rabbit IgG (ab150078, abcam, UK) and DAPI, separately. The subcellular localization of Nrf2 was visualized using inverted fluorescence microscope (magnification, ×200) (Nikon 80i, Japan).

### Western Blot Analysis

Proteins from cells or liver tissues were separated by 10% sodium dodecyl sulfate-polyacrylamide gel electrophoresis (SDS-PAGE) gel and electrophoretically transferred to polyvinylidene difluoride (PVDF) membranes (162-0177, Bio-Rad, Hercules, CA, USA). After being blocked by 5% non-fat dry milk (232100, BD, MD, USA) for 2 h at room temperature, the protein bands were incubated with specific antibodies against Nrf2, GAPDH (bsm-0978M, bisso, Beijing, China), Lamin B1 (13435, CST, Danvers, MA, USA), Cleaved Caspase-3 (9664,CST), Cleaved Caspase-9 (20750, CST), Cytochrome C (Cyto C, 11940, CST), p53 (ab26, abcam), Bcl2, bax, Keap-1 (Kelch-like ECH-associated protein 1) (10503-2-AP, Proteintech), HO-1 (16396-1-AP, Proteintech), NQO-1 (67240-1-IG, Proteintech) overnight at 4°C. All primary antibodies diluted 1:1000.After being rinsed thrice with TBST at 5-min intervals, the membranes were incubated with horseradish peroxidase-labeled goat anti-rabbit IgG (ab6697, abcam, UK) or goat anti-mouse IgG (ab205719, abcam) for 2 h at room temperature. Immunoblots were visualized using the Immobilon Western Chemiluminescent HRP Substrate (WBKLS0500, Millipore Corporation, Billerica, MA, USA) with Bio-Rad Chemi Doc MP. All immunoblot analysis data were performed in triplicate. Densitometry analysis was performed using Image J 6.0 software (National Institutes of Health, Bethesda, MD, USA).

### Caspase-3,-9 Activity Assay

The LO2 cells or liver homogenate were incubated in ice-cold lysis buffer for 20 min, then centrifuged at 10,000g for 2 min. Caspase-3, -9 Colorimetric Assay Kit (C1115 and C1157) was used to detect activity of Caspase-3, -9 according to the manufacturer’s manipulations and absorbance at 405 nm was measured with a microplate reader. The related results were showed as a ratio to control.

### Measurement of Mitochondrial Membrane Potential

After the cells were treated, conducted as described above, LO2 cells were collected and incubated with 10 μg/ml JC-10 (40752ES60, Yeasen, shanghai, china) for 30 min at 37°C in the dark. JC-10 monomers emit green fluorescence at 527 nm in apoptotic and unhealthy cells with low membrane potential. The cells were then washed twice with PBS, and the fluorescence intensity was observed under a fluorescence microscope (magnification, ×100) (Nikon 80i, Japan). ΔΨm was represented by the ratio of JC-10 red fluorescence (aggregated form) to green fluorescence (monomeric form).

### Annexin V-FITC/Propidine Iodide Double Staining Analysis

After the cells were treated, three replicate wells were used for each experimental condition. Then washed twice in cold PBS (4°C), cells apoptosis were performed using Annexin V-FITC/Propidine Iodide (PI) Apoptosis Detection Kit (40302ES60, Yeasen). The result of AV/PI-positive cells was evaluated with a flow cytometer (BD Accuri™ C6, USA).

### Statistical Analysis

The data are presented as the Mean ± SEM. The comparisons among several groups were performed with a one-way ANOVA using SPSS 21. Data that did not demonstrate a normal distribution were analyzed using a Mann-Whitney U test. P<0.05 was considered to be significant.

## Result

### UDCA Ameliorated As(III)-Induced Liver Injury in Mice

**Figure 1:**
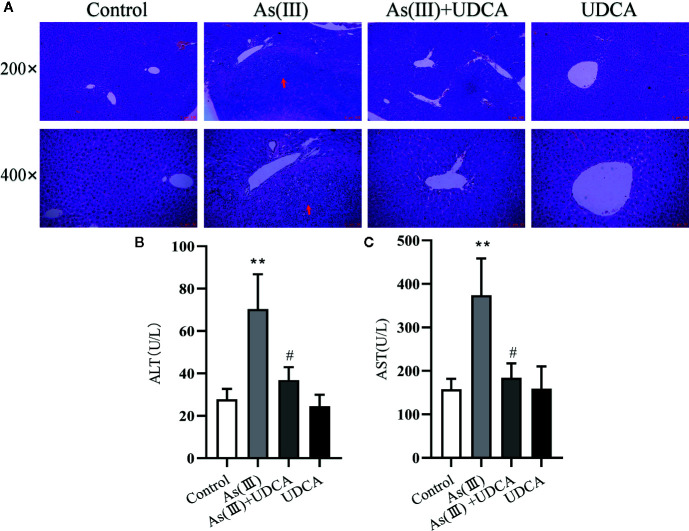
Ursodeoxycholic acid (UDCA) ameliorated As(III)-induced liver injury in mice. **(A)** Effects of UDCA on hepatic histopathologic changes in As(III)-treated mice. Representative images of hematoxylin & eosin (HE) staining were used to detect liver histopathologic changes. Scale bar = 100 µm (up, magnification 200×), Scale bar = 50 µm (down, magnification 400×). Prominent inflammation and tissue damage (indicated by red arrows) in As(III)-treated mice liver. Effects of crocetin on activities of **(B)** alanine aminotransferase (ALT) and **(C)** aspartate aminotransferase (AST) in each group. Data are presented as Mean ± SEM (n = 8). Significant differences are shown as **P < 0.01, compared with the control group. ^#^ *P <* 0.05, compared with the As(III) group.

The body mass and liver index of mice are shown in **Table 1**. Liver weight and Liver weight index increased in As(III) treated group when compared to Control group (*P <* 0.05), UDCA (30 mg/kg) had no effect on the decreased body weight and the increased Liver weight and Liver weight index in As(III)-induced mice. In order to investigate the protection of UDCA in the As(III)-induced liver injury, we assessed histopathological changes of liver and the serum ALT, AST levels. As shown in **Figure 1A**, histological analysis by H&E staining of the liver suggested livers from the Control group and UDCA alone group displayed regular cell distribution and normal lobular architecture. The liver tissues from As(III)-treated mice showed obvious pathological changes, including hepatocyte steatosis, apoptosis, disorganization of parenchyma and excessive inflammatory cells infiltration. However, these effects were ameliorated by UDCA treatment. The infiltration of inflammatory cells into the liver was markedly reduced and few lipid droplets were found in As(III)+UDCA-treated mice. In addition, As (III) group had remarkably increased levels of serum ALT and AST (*P <* 0.01) (**Figures 1B, C** **)** compared to those in the negative control group, whereas UDCA treatment significantly diminished the levels of ALT and AST in the serum (*P <* 0.05). These results showed that UDCA play a protective role in As(III)-induced liver injury in mice.

**Table 1:** Effect of fucoxanthin on body weight and liver index of control and experimental animals.

Group	Body weight		Liver weight (g)	Liver weight index (%)
	Initial (g)	Final (g)		
Control	20.08 + 1.15	22.05 + 1.30	0.96 + 0.04	4.35 + 0.32
As(III)	20.05 + 0.68	21.43 + 2.25	1.16 + 0.10*	5.46 + 0.59*
As(III)+UDCA	20.07 + 0.66	21.764 + 1.64	1.06 + 0.12	4.86 + 0.49
UDCA	20.00 + 0.88	21.73 + 1.26	1.06 + 0.11	4.89 + 0.73

Values are measured as mean ± SEM *Significant when compared to the control group.

### UDCA Alleviated As(Ⅲ)-Induced Liver Oxidative Stress in Mice

**Figure 2:**
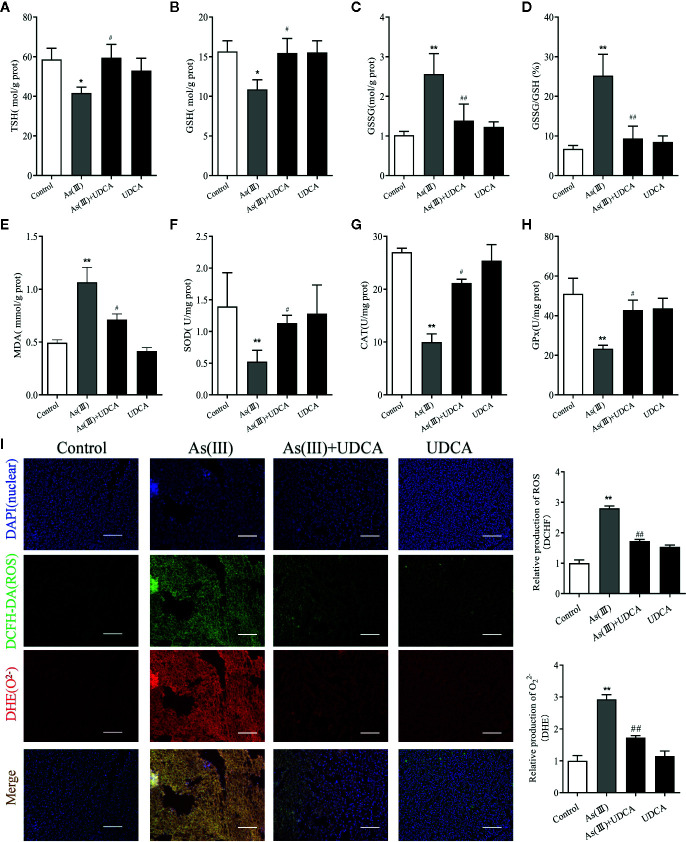
Ursodeoxycholic acid (UDCA) alleviated As(III)-induced liver oxidative stress in mice. Hepatic **(A)** Total sulfhydryl (TSH), **(B)** glutathione (GSH), **(C)** GSSG levels, **(D)** rate of GSSG/GSH, **(E)** malondialdehyde (MDA), **(F)** superoxide dismutase (SOD), **(G)** catalase (CAT), and **(H)** GSH peroxidase (GSH-px) activity were tested in mouse after 7 days of treatment using enzymatic recycling assay. Data are presented as Mean ± SEM (n = 6). **(I)** Effect of UDCA on As(III)-induced reactive oxygen species (ROS) and O_2_^2-^ from mice of different groups after 7 days of treatment. DCFH-DA and dihydroethidium (DHE)-stained liver tissue sections were observed under fluorescence microscope. Scale bar: 100 μm (magnification 200×). DCFH-DA and DHE band intensities scanned by densitometer. Data are presented as Mean ± SEM (n = 3). Significant differences are shown as **P < 0.01, compared with the control group. ^#^ *P <* 0.05, ^##^p< 0.01, compared with the As(III) group.

To assess the anti-oxidative effects of UDCA, the levels of MDA, GSH, CAT, and SOD were measured in mouse liver tissue lysate. We observed that the level of TSH and GSH was negatively affected by As(III) and its oxidized form GSSG level and GSSG/GSH ratio got amplified (*P <* 0.01) (**Figures 2A–D**). In addition, a significant (*P <* 0.05) increase level of MDA and decrease activities of SOD, CAT, and GSH-Px was found in As(III) group compared with Control group (*P <* 0.01) (**Figures 2E–H**). However, UDCA significantly increase GSH, TSH and decrease GSSG level and GSSG/GSH ratio compared to As(III)-treated group alone (*P <* 0.05 or *P <* 0.01) (**Figures 2A–D**), the level of MDA got reversed and CAT, GSH-Px, and SOD activities were improved in the As(III)+UDCA groups (*P <* 0.05 or *P <* 0.01) (**Figures 2E–H**). As(III) induced cell oxidative stress was evaluated by measuring the ROS level produced in mice liver (**Figure 2I** **)**. To investigate the generation of ROS, fluorescent double staining of DCFH-DA and DHE showed that As(III) exposure strongly increased DHE intensity of ROS generation in mice liver (*P <* 0.01). After treatment of UDCA, the generation of ROS and O_2_^2-^ were decreased strongly (*P <* 0.01). No significant change was noted in the UDCA alone treated mice. While UDCA significantly reduced the levels of lipid peroxidation and enhanced activities of antioxidant enzymes in comparison with the As(III) group.

### UDCA Inhibited As(III)-Induced Liver Apoptosis in Mice

**Figure 3:**
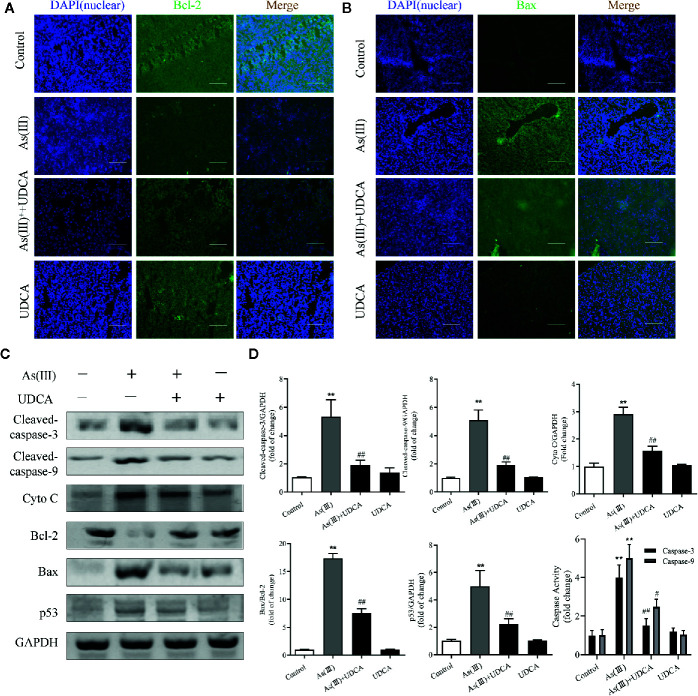
Ursodeoxycholic acid (UDCA) inhibited As(III)-induced liver apoptosis in mice. Representative fluorescence images of **(A)** Bcl-2 and **(B)** Bax staining for liver tissue sections obtained from mice of different groups after 7 days of treatment, whereas the cell nuclei were stained with DAPI. Liver tissue section cells were observed under fluorescence microscope. Scale bar: 100 μm (magnification 200×). **(C)** Hepatic levels of Cleaved Caspase-3, and 9, Cyto C, p53 and rate of Bax/Bcl-2 were analyzed by western blotting with specific antibodies. Representative blots were shown. GAPDH was used as loading control. Data are presented as Mean ± SEM (n = 3). **(D)** Hepatic activities of Caspase-3, and 9 were also quantified. Data are presented as Mean ± SEM (n = 6). Significant differences are shown as **P < 0.01, compared with the control group. ^#^ *P <* 0.05, ^##^ *P <* 0.01, compared with the As(III) group.

To further investigate the role of UDCA in As(III)-induced liver cells dysfunction, the expression of apoptosis related proteins was detected. As shown in **Figures 3A, B**, the apoptosis-regulatory protein Bcl-2 and Bax detected by Immunofluorescence showed that UDCA effectively increased the protein fluorescence intensity of Bcl-2 and reduced protein fluorescence intensity of Bax compared with As(III)-exposed group. Additionally, this was confirmed by western blot analysis. The protein levels of Cleaved-Caspase-3, 9, p53, Cyto C, the ratio of Bax/Bcl-2 and the activity of Caspase-3, 9 were significantly increased in As(III) group (*P <* 0.01) compared with the levels in the Control group, However, the levels of apoptosis-regulatory protein was decreased significantly in the UDCA+As(III) group (*P <* 0.05 or *P <* 0.01) (**Figure 3C**). There were no significant difference in UDCA alone administered group.

### UDCA Induced Nrf2 Activation on As(Ⅲ)-Challenged Hepatotoxicity in Mice

**Figure 4:**
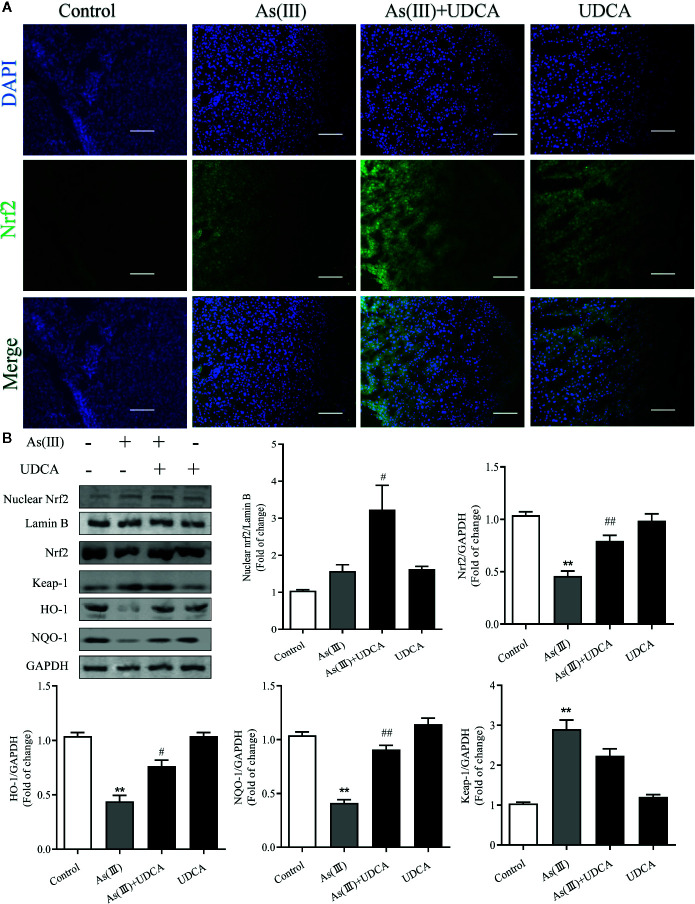
Ursodeoxycholic acid (UDCA) induced Nrf2 activation on As(III)-challenged hepatotoxicity in mice. Representative fluorescence images of **(A)** Nrf2 staining for liver tissue sections obtained from mice of different groups after 7 days of treatment, whereas the cell nuclei were stained with DAPI. Liver tissue section cells were observed under fluorescence microscope. Scale bar: 100 μm (magnification 200×). **(B)** Nuclear Nrf2 protein levels were determined by densitometric analysis and normalized to the Lamin B signal. Nrf2 antioxidant-related proteins, including Nrf2, Keap-1, HO-1, NQO1 in total lysates were also analyzed by were analyzed by western blotting with specific antibodies. Representative blots were shown. Lamin B and GAPDH was used as loading control. Data are presented as Mean ± SEM (n = 3). Significant differences are shown as **P < 0.01, compared with the control group. ^#^ *P <* 0.05, ^##^ *P <* 0.01, compared with the As(III) group.

Nrf2 is the key protein regulating downstream antioxidant expression, and its nuclear levels were always used to determine Nrf2 nuclear translocation (Reisman et al., 2009). To investigate whether As(III) or UDCA could affect Nrf2 translocation, therefore, immunofluorescence assay and western blot were performed to detect Nrf2 level in the plasma and nucleus of liver. Through immunofluorescence staining, the nucleus (blue) stained with DAPI (**Figure 4A**, upper image), the Nrf2 (green)-stained fluor-conjugated secondary antibody (middle image), and the merged image of As(III), UDCA or both of them-treated liver cells showed the nuclear location of Nrf2 protein. As(III) and UDCA slightly increased the intranuclear level of Nrf2, but As(III)+UDCA exerted this effect better. This was confirmed by western blot analysis (**Figure 4B**), showing higher levels of Nrf2 in the nucleus of the As(III)-treated group as compared to the cells treated in the Control group. This intranuclear level increase further under the UDCA-treated conditions in As(III)-induced mice, so the nuclear translocation would be more obvious (*P <* 0.05). As shown in **Figure 4B**, compared with the Control group, the protein expression of Keap-1 was increased (*P <* 0.05). A contrary tendency was observed for NQO-1 and HO-1 protein expression, which were downstream Nrf2 antioxidant in the As(III) treatment group. Furthermore, Nrf2, NQO-1 and HO-1 protein expressions were elevated in the As(III)+UDCA group (*P <* 0.05 or *P <* 0.01), however, Keap-1 protein expression was decreased but did not show a significant difference. These results suggest that UDCA plays a regulatory role in As(III)-induced oxidative stress in mice liver tissues.

### UDCA Mitigated As(III)-Induced Cytotoxicity in LO2 Hepatocytes

**Figure 5:**
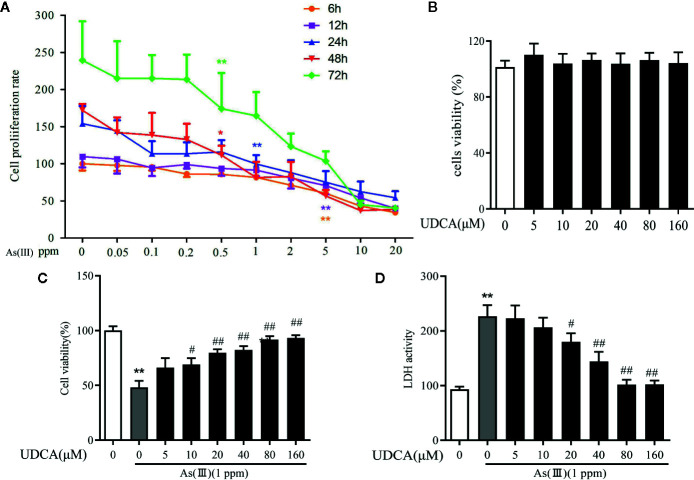
Ursodeoxycholic acid (UDCA) mitigated As(III)-induced cytotoxicity in LO2 hepatocytes. **(A)** LO2 cells cells were stimulated with 0.05 to 20 ppm As(III) and cell viability was evaluated at different time intervals. **(B)** The cells were treated with UDCA of escalating concentrations for 24 h, followed by assessment of cell viability. **(C)** The cells were pre-treated with 0 to 120 μM of UDCA for 24 h, and then incubated with 1 ppm As(III) for another 24 h by using CCK-8 assay. **(D)** Lactate dehydrogenese (LDH) leakage into the culture medium of LO2 cells with/without 1 ppm As(III) in the presence or absence of treatment pretreatment with 0 to 120 μM UDCA measured by non radiative cytotoxicity assay kit. Data are presented as Mean ± SEM (n = 6). Significant differences are shown as **P < 0.01, compared with the control group. ^#^ *P <* 0.05, ^##^ *P <* 0.01, compared with the As(III) group.

To measure the potential cytotoxicity of As(III), the effects on the viability of LO2 cells were assessed by the CCK-8 assay. The viability of LO2 cells was dramatically decreased in a dose and time-dependent manner as As(III) concentrations were increased from 0.01 to 10 ppm for 6 h to 72 h. As(III) at 1 ppm incubated for 24 h caused cell viability decrease to 46.5% (**Figure 5A**) (*P <* 0.01). Therefore, in subsequent procedures, treatment with 1 ppm As(III) for 24 h was used to establish the oxidative stress model of LO2 cells. To explore the cytotoxicity of UDCA on normal LO2 cells, We found that treated cells with 0 to 160 μM of UDCA. As shown in **Figure 5B**, there was no obvious change on cell viability of LO2 cells after treatment of different doses of UDCA. Next, we investigated the protective effects of UDCA on LO2 cells viability of As(III)-induced LO2 cells. As shown in **Figure 5C**, As(III) significantly suppressed cell growth compared with the control group. However, UDCA ameliorated the toxic effect induced by As(III) on LO2 cells in a concentration-dependent manner. In addition, after treatment for 24 h, a significant increase in LDH release were observed in LO2 cells treated with 1ppm As(III) compared with control (*P <* 0.01). Pretreatment with 20 to 160 μM of UDCA significantly decreased LDH activity (*P <* 0.05), which indicated that UDCA could alleviate acute liver injury induced by As(III) administration. Pretreatment with UDCA (for 24 h) at a dose of 20 μM was found to be the optimum protective dose against As(III) induced loss of LO2 cells viability (**Figure 5D**). With this in mind, 20 μM of UDCA was used in the subsequent experiments.

### UDCA Attenuated As(III)-Induced Cell Death *via* Nrf2 Activation

**Figure 6:**
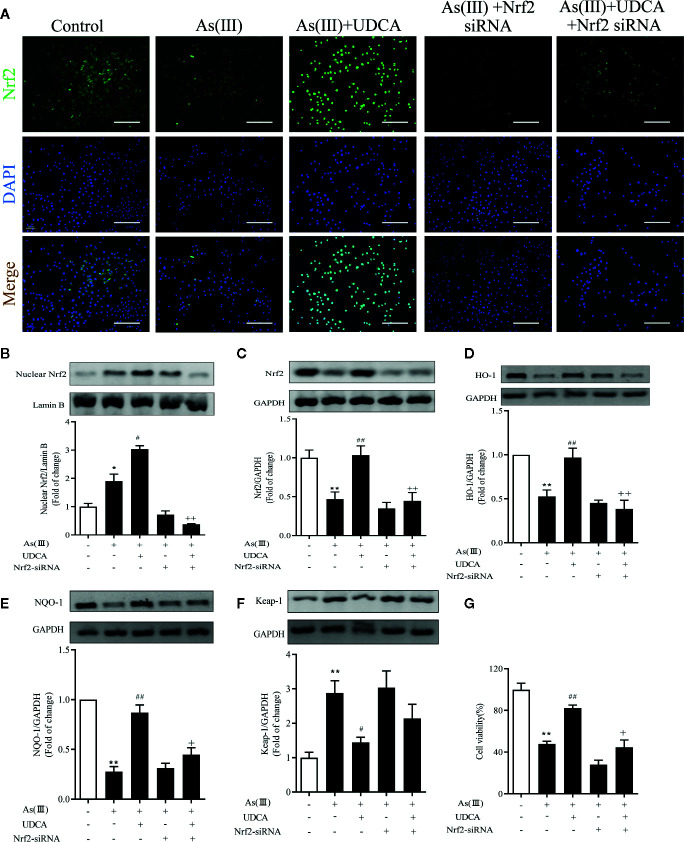
Ursodeoxycholic acid (UDCA) attenuated As(III)-induced LO2 hepatocytes death *via* Nrf2 activation. LO2 cells were transfected with NC siRNA or Nrf2 siRNA for 48 h, and then incubated with UDCA, and then subjected to As(III) stimulation. **(A)** After drug treatment, the cells were stained for Nrf2 subunit, whereas the cell nuclei were stained with DAPI. The cells were imaged under fluorescence microscopy. Scale bar: 100 μm (magnification 200×). **(B)** Protein expressions of Nrf2 proteins in nuclear extracts, Lamin B was used as a protein control to normalize volume of protein expression. Nrf2 antioxidant-related proteins, including **(C)** total Nrf2, **(D)** HO-1, **(E)** NQO1, **(F)** Keap-1 in total lysates were also analyzed by were analyzed by western blotting with specific antibodies. Representative blots were shown. Lamin B and GAPDH was used as loading control. Data are presented as Mean ± SEM (n = 3). **(G)** Cell viability was evaluated by CCK-8. Data are presented as Mean ± SEM (n = 6). Significant differences are shown as **P < 0.01, compared with the control group. ^#^ *P <* 0.05, ^##^ *P <* 0.01, compared with the As(III) group. ^++^ *P <* 0.01, ^+^ *P* < 0.05, compared with the As(III)+UDCA group *P < 0.05.

To explore the effects of Nrf2 knockdown on the inhibition of As(III) stimulated cytotoxicity activation by UDCA treatment, LO2 cells were transfected with 40 nM Nrf2 siRNA or NC siRNA and then subjected to UDCA treatment. There was no obvious change on cell viability of LO2 cells after transfection with Nrf2 siRNA or NC siRNA in the absence or presence of UDCA (**Supplementary Figure 1A**). Compared with the NC siRNA transfected cells, nucleus and total Nrf2 were reduced in Nrf2-siRNA-transfected cells (*P <* 0.01). Furthermore, UDCA could not rescue intracellular Nrf2 levels in siRNA-transfected cells (*P <* 0.01) (**Supplementary Figure 1B, C**). The nuclear Nrf2 expression was observed using immunofluorescence and western blot (**Figures 6A, B** **)**. These results revealed that As(III) caused a significant decrease in total Nrf2 protein expression and increase nuclear Nrf2 level in LO2 cells, Nuclear Nrf2 expression was stronger in the As(III)+UDCA group compared to the As(III) group in LO2 cells (*P <* 0.01). UDCA pretreatment also increased UDCA treatment was found to increase the total Nrf2, HO-1, NQO-1 level and decrease Keap-1 level comparison with that of the As(III) group in LO2 cells (*P <* 0.05). Meanwhile, After transfected with Nrf2-siRNA, the As(III)+UDCA groups in Nrf2-siRNA-transfected cells showed no statistically significant difference (**Figures 6C–F**). To further confirm the protective effect of Nrf2. CCK-8 was used to reveal cell viability. Compared to As(III) stimulated control cells, knockdown of Nrf2 was found to aggravate cell death. In Nrf2-siRNA-transfected cells, UDCA treatment failed to decrease As(III)-stimulated increase in cell death (*P <* 0.01) (**Figure 6G**).

### UDCA Alleviated As(III)-Induced LO2 Hepatocytes Oxidative Stress *via* Nrf2 Activation

**Figure 7:**
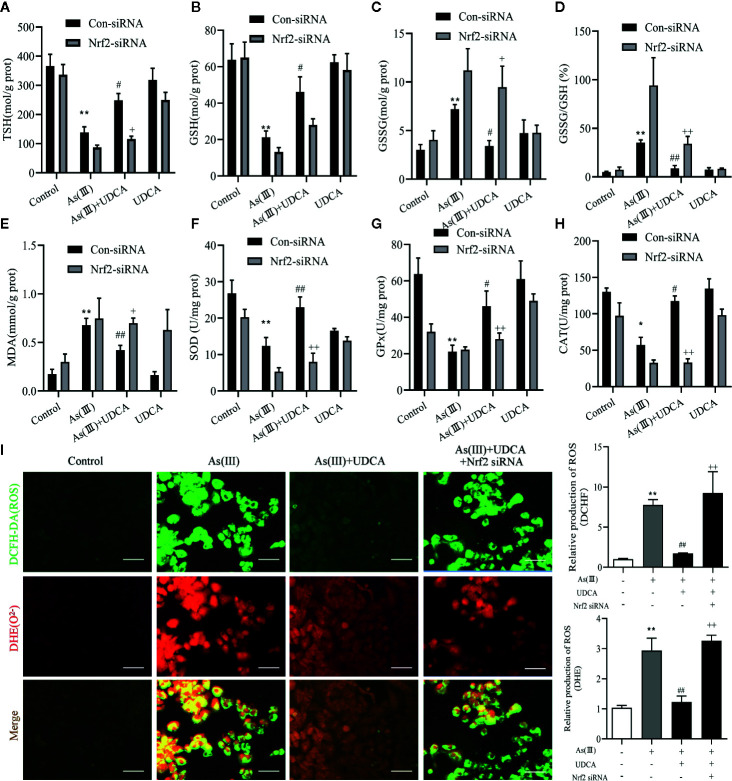
Ursodeoxycholic acid (UDCA) alleviated As(III)-induced LO2 hepatocytes oxidative stress *via* Nrf2 activation. LO2 cells were transfected with Nrf2 siRNA for 48 h, and then incubated with UDCA, and then subjected to As(III) stimulation. **(A)** Total sulfhydryl (TSH), **(B)** glutathione (GSH), **(C)** GSSG levels, **(D)** rate of GSSG/GSH, **(E)** malondialdehyde (MDA), **(F)** superoxide dismutase (SOD), **(G)** catalase (CAT) and **(H)** GSH peroxidase (GSH-px) activity were tested in LO2 hepatocytes using enzymatic recycling assay. **(I)** Effect of UDCA on As(III)-induced ROS and O_2_^2-^ from LO2 cells of different groups. DCFH-DA and DHE-stained cells were observed under fluorescence microscope. Scale bar: 100 μm (magnification 200×). DCFH-DA and DHE band intensities scanned by densitometer. Data are presented as Mean ± SEM (n = 3). Significant differences are shown as **P < 0.01, compared with the control group. ^#^ *P <* 0.05, ^##^ *P <* 0.01, compared with the As(III) group. ^++^ *P <* 0.01, ^+^ *P* < 0.05, compared with the As(III)+UDCA group *P < 0.05.

Increased oxidative stress has been reported in As(III)-induced in LO2 cells injury. In **Figure 7**, the results show that the antioxidant related substances (TSH, GSH, SOD, CAT, and GSH-Px) in the cells homogenate marked an decrease in the As(III)-induced LO-2 cells, In the levels of GSSH, GSSG/GSH ratio, MDA, ROS were found to be high in As(III)-treated groups (*P <* 0.01) compared with the Control group. whereas in the As(III)+UDCA received group showed the significant reduction while compared with As(III)-induced LO2 cells (*P <* 0.05 or *P <* 0.01). In Nrf2-siRNA-transfected cells, UDCA treatment failed to decrease As(III)-stimulated increase in level of GSH, TSH, MDA, ROS and the activities of CAT, GSH-Px, and SOD (*P <* 0.05 or *P <* 0.01).

### UDCA Mitigated As(III)-Induced LO2 Hepatocytes Apoptosis *via* Nrf2 Activation

**Figure 8:**
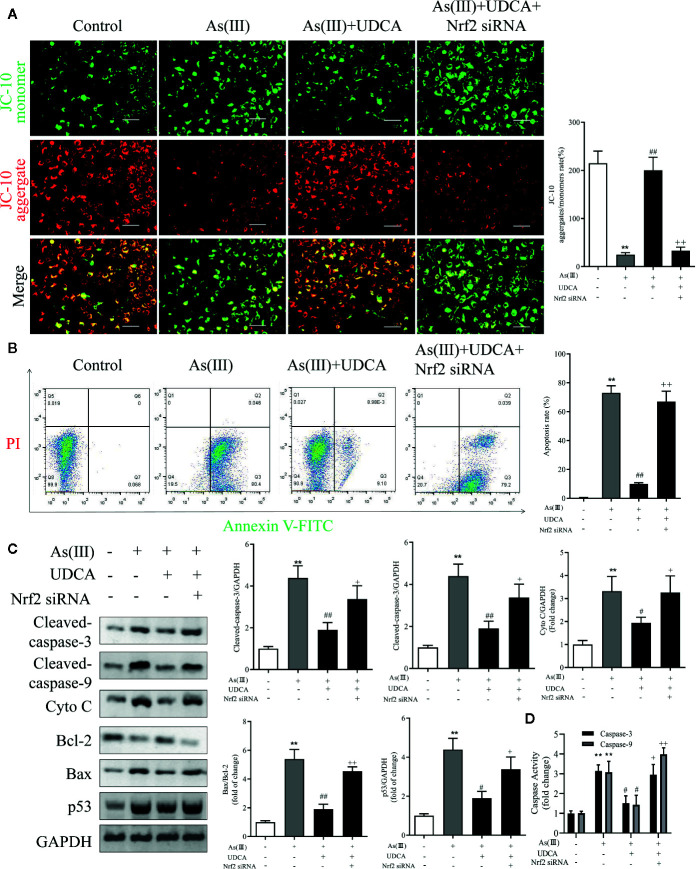
Ursodeoxycholic acid (UDCA) mitigated As(III)-induced LO2 hepatocytes apoptosis *via* Nrf2 activation. LO2 cells were transfected with Nrf2 siRNA for 48 h, and then incubated with UDCA, and then subjected to As(III) stimulation. **(A)** LO2 cells was evaluated by JC-10 staining under a fluorescence microscope. Scale bar: 100 μm (magnification 200×). Red and green fluorescence scanned by densitometer. Data are presented as Mean ± SEM (n = 3). The ratio of red to green fluorescence in cells were calculated **(B)** Induction of apoptosis was measured by Annexin-V/PI double staining was detected by flow cytometry. The apoptosis rate of Annexin-V/PI double-stained LO2 cells cells was calculated from flow cytometric analysis. **(C)** Hepatic levels of Cleaved Caspase-3, and 9, Cyto C, p53 and rate of Bax/Bcl-2 were analyzed by Western blotting with specific antibodies. Representative blots were shown. GAPDH was used as loading control. Data are presented as Mean ± SEM (n = 3). **(D)** Hepatic activities of Caspase-3, and 9 were also quantifified. Data are presented as Mean ± SEM (n = 6). Significant differences are shown as **P < 0.01, compared with the control group. ^#^ *P <* 0.05, ^##^ *P <* 0.01, compared with the As(III) group. ^++^ *P <* 0.01, ^+^ *P* < 0.05, compared with the As(III)+UDCA group.

To determine the effect of As(III) and UDCA on mitochondrial dysfunction, mitochondrial membrane potential (MMP) of LO2 cells was measured in each experimental groups. Under stressed conditions mitochondrial membrane is depolarized and JC-10 stained cells show shift from red to green fluorescence. As shown in **Figure 8A**, As(III) induced a significant increase in cell number with depolarized mitochondria (green fluorescence/red fluorescence) (*P <* 0.01), However, the proportion of MMP-depolarized cells gradually decreased in cells treated with UDCA before exposure to As(III) (*P <* 0.01). These results indicate that UDCA alleviated the As(III)-induced decrease of MMP depolarization in LO2 cells, thereby protecting LO2 cells from oxidative stress-induced mitochondrial damage be reversed by the treatment of UDCA. To examine whether UDCA inhibited As(III)-induced LO2 cells apoptosis, Annexin V-FITC/PI staining was used to determine the apoptosis of LO2 cells. As shown in **Figure 8B**, the flow cytometry analysis of LO2 cells showed that the cell population tended to shift from viable to apoptotic on treatment with As(III) (*P <* 0.01). However, after treatment with UDCA, the apoptotic cells were found lower in LO2 cells as compared to the As(III) group (*P <* 0.01). In Nrf2-siRNA-transfected cells, UDCA treatment failed to decrease As(III)-stimulated increase in MMP depolarization and apoptosis rate (*P <* 0.05 or *P <* 0.01). In addition, after As(III)+UDCA exposure, the protein levels of Cleaved-caspase-3, 9, p53, Cytochrome C, the ratio of Bax/Bcl-2 and the activity of Caspase-3, 9 were significant reduction while compared with As(III)-induced LO2 cells. As expected, which could be abolished by Nrf2 siRNA (*P <* 0.01) (**Figures 8C, D** **)**.

## Discussion

Oxidative stress is currently the most widely accepted and studied mechanism of arsenic toxicity (Flora, 2011; Sharma et al., 2017; Okereafor et al., 2020). The abnormal increase of hepatocytes apoptosis is one of the important pathological mechanisms of arsenic-induced hepatotoxicity. Therefore, the inhibition of hepatocytes apoptosis is one of the indexes to investigate the efficacy of liver protective drugs (Kim et al., 2004; Ozaki, 2019). UDCA is a type of hydrophilic bile acid extracted from animal bile with a wide range of biological functions. The present results demonstrated that UDCA exerts many medicinal benefits by preventing the several agents induced oxidative injury, through a direct antioxidant effect or a stimulate anti-oxidant enzymes, thereby ameliorating hepatocytes damage (Makino and Tanaka, 1998; Perez and Briz, 2009; Ali et al., 2020). However, no previous studies have investigated the therapeutic effect of UDCA on As(III)-induced liver damage. In this study, we used an As(III)-induced hepatic injury model to investigate the hepatoprotection of UDCA *in vivo* and *in vitro*. Therefore, the results showed that UDCA can exert protective effects against As(III)-induced cellular oxygen species accumulation and apoptosis in hepatocytes.

As(III)-induced hepatoxicity was evaluated by and histological and biochemical assays. Histological analysis indicated that UDCA attenuated hepatic pathologic changes, including infiltration of inflammatory cells and disintegration with necrosis. These results demonstrated that UDCA could alleviate the damage of hepatocytes. Meanwhile, obtained results showed As(III) markedly elevated the levels of serum biochemical indicators ALT and AST were higher, which is consistent with previous studies (Ling et al., 2017). UDCA treatment remarkably inhibited elevations of ALT and AST, which demonstrated that UDCA could alleviate As(III)-induced hepatotoxicity *in vivo* (**Figure 1**). To further confirm our hypotheses and investigate the potential mechanisms, we screened a concentration of As(III) (1ppm, equivalent to 6.67 μM As_2_O_3_) to construct an *in vitro* liver cell injury model. At the same time, we found that As(III) in dose and time dependently decreased the viability of hepatocytes and significantly increased LDH activity in LO2 cells. We showed that UDCA could enhance cell viability and reduce LDH activity, although treatment of UDCA alone did not lead to significant changes in viability of hepatocytes (**Figure 5**). It was demonstrated that UDCA could decrease As(III)-induced cell death *in vitro*. Besides, The protective effect of UDCA is significantly inhibited in As(III) induced Nrf2-silenced LO2 cells (**Figure 6**). Therefore, the activation of Nrf2 might play a critical role in the functional effect of UDCA, and we boldly speculated that UDCA ameliorated As(III)-induced oxidative stress partly dependent on the activation of Nrf2.

Oxidative stress is a consequence of an imbalance between generation and elimination of ROS (Yan et al., 2019). Consistent with this notion, As(III) induced intracellular ROS accumulation in hepatocytes as evidenced by DCFH-DA and DHE fluorometry *in vivo* and *in vitro* (**Figures 2**, **7**). These results demonstrated that the generation of ROS and O^2-^ was found evidently aggrandized in hepatocytes treated with As(III). Moderate intercellular ROS contribute to Nrf2 activation to exert protective function through inducting its target antioxidant genes (Tebay et al., 2015). In turn, Nrf2 defense system activation relieves oxidative stress damage by modulating ROS and inflammatory process (Shang et al., 2017). As an antioxidant, UDCA has been shown to inhibit ROS generation and activate Nrf2 signaling pathway in hepatocytes (Okada et al., 2008; Arisawa et al., 2009). In the presence of UDCA, the augmented ROS production induced by As(III) was evidently abated, although treatment of UDCA alone did not lead to significant changes in ROS level.

GSH is one of the effective antioxidants in cells (Silva-Adaya et al., 2020). As(III) also has a strongly binding ability of vicinal thiols or biological ligands containing cysteine residues *in vitro* and *in vivo*, while the elimination of ROS needs substances containing sulfur groups. In the present study, treatment with As(III) inhibited the antioxidant effect of GSH, reduced the TSH and increased the GSSG, lead to the accumulation of ROS. Interestingly, UDCA treatment markedly declined these oxidative stress parameters. Some study found that treatment of UDCA alone lead to significant changes in GSH levels, although this finding is in agreement with our results in some ways (Zhang et al., 2017; Wang et al., 2019). Under normal physiological conditions, Nrf2 maintains a low expression level in the cytoplasm in combination with the inhibitory protein Keap-1 and thus prevents Nrf2 transcription. However, upon stimulation with various inducers such as reactive oxygen species, Keap-1 in the cytoplasm dissociates from Nrf2. As(III) and Excessive ROS production results in the change of Keap-1 conformation by modifying cysteine residues (Cys257, Cys273, Cys288, Cys293) (Eggler et al., 2005). In our results, As(III) promotes the nuclear transfer of Nrf2 in the hepatocytes *in vivo* and *in vitro* (**Figures 4**, **6**), however, this kind of defense is limited. This dissociated free Nrf2 is dissociated and transferred to the nucleus, which binds to an antioxidant response element (ARE) to initiate the transcription of downstream antioxidant enzymes, binomial detoxifying enzymes, including SOD, CAT, NQO-1, and HO-1, completing the antioxidant effect. The increase of these factors elevated the antioxidant capacity of cells. The upswing in the activities of SOD and CAT which are the first line of cellular defense as well as in the GSH level and activities of GSH-dependent enzymes namely GSH-Px (Perez et al., 2006; Battogtokh et al., 2018). Obtained results showed markedly decreased activities of antioxidant enzymes such as SOD, CAT, GSH-Px in hepatocytes exposure to As(III). MDA, are presentative lipid peroxidation product, indirectly reflects the degree of hepatocytes damage (Moniruzzaman et al., 2018). In addition, MDA levels increased significantly after As(III) poisoning, which indicated that the formation of peroxides was enhanced in response to toxicity. These results seem to be in agreement with the previous findings (Hu et al., 2020; Huo et al., 2020). However, UDCA promoted activity of SOD, CAT and GSH-Px and inhibited levels of MDA, ROS to the best level in hepatocytes, but it was still slightly lower than the Control. Nrf2 knockdown partially abrogated those effect (Zhong et al., 2018). Additionally, we revealed that UDCA promoted the nuclear translocation of Nrf2 and increased the levels of HO-1, NQO-1 in hepatocytes both *in vivo* and *vitro*, accompanied by a reduction of As(III)-provoked oxidative stress and subsequent hepatotoxicity. Interestingly, we found that the protective effects of UDCA on oxidative stress were blocked when Nrf2 was silenced. As expected, UDCA lost its effects in increasing the expression of nucleus and total Nrf2 in As(III)-induced Nrf2 knockdown cells.

**Figure 9:**
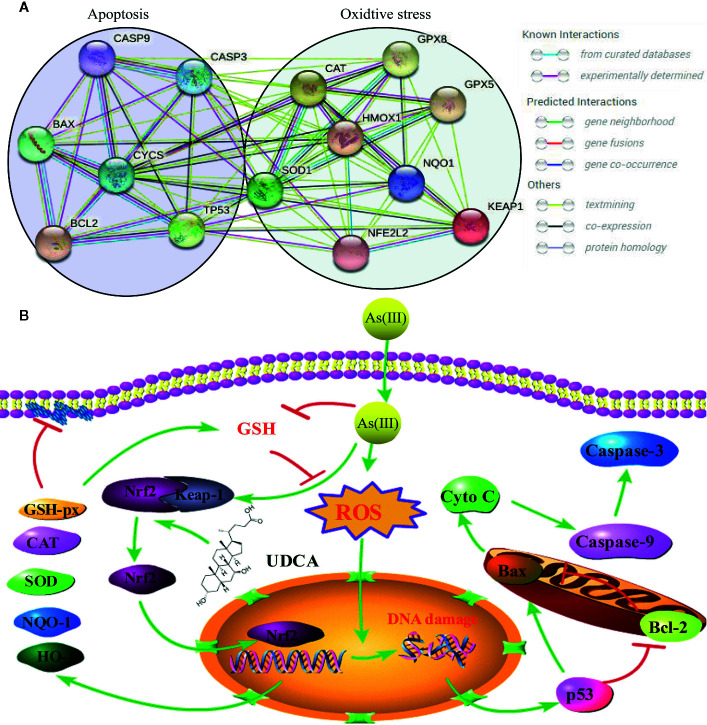
Schematic presentation probable protective mechanism of Ursodeoxycholic acid (UDCA) against As(III)-mediated hepatic injury by activating the Nrf2 pathway. **(A)** Protein-protein interaction (PPI) network between Nrf2-mediated oxidative stress pathway related proteins (SOD1, CAT, Gpx, HO-1, NQO-1, Keap1, Nrf2) and p53 mediated apoptosis pathway related proteins (Caspase-3, 9, Bax, Bcl-2, p53, CYCS) by STRING. **(B)** The green arrow indicated the pathological events involved within As(III)-exposed LO2 cells. The red lines denoted the activity restricted by UDCA.

The Nrf2 pathway is a major signal transduction pathway responsible for mitochondrial dysfunction and apoptosis (Reisman et al., 2009; Choi and Jeon, 2020). Excessive production of ROS can also cause lipid peroxidation damage of mitochondrial membrane and decrease MMP, so the effects of As(III) on mitochondrial function were evaluated by measurement of MMP by JC-10. The results showed that pretreatment of LO2 cells with UDCA could antagonize the lipid peroxidation reaction of As(III) to mitochondrial membrane and maintain the MMP, which could be abolished by Nrf2 siRNA. The decrease of MMP in mitochondria is considered to be the first event in the process of apoptosis cascade. Mitochondrial dissipation could promote the release of Cyto C, activate Caspase protease family and cause cell apoptosis cascade reaction. As(III) induced apoptosis plays the most important role in the pathogenesis of arseniasis (Zhang et al., 2017; Wang et al., 2019). At presently, the cells apoptosis mainly includes three pathways: mitochondrial pathway, death receptor pathway, endoplasmic reticulum pathway. It was notoriously that As(III) induced necrotic and apoptotic cell death that is dependent on the ROS mediate mitochondrial intrinsic pathway (Perez et al., 2006; Yan et al., 2019). Caspase-3, 9 is one of the most important components of Caspase family that cause apoptosis (Kim et al., 2015). In this study, UDCA decreased Caspase-3, 9 activity and Cleaved-Caspase-3, 9 protein expression in As(III)-induced hepatocytes *in vivo* and *vitro*. As(III) exposure stimulated excessive ROS generation, which seems to play a critical role in promotion the activity of p53 pathway (Sena and Chandel, 2012; Alamolhodaei et al., 2015). p53 is a tumor suppressor protein that promotes the apoptotic process (Jiang and Rusling, 2019; Silva-Adaya et al., 2020) and directly regulate the activity of the pro-apoptotic protein Bax, which further facilitates mitochondrial membrane permeabilization and apoptosis, especially the expression of several members of the Bcl-2 family (Binju et al., 2019). The nuclear fraction of Nrf2 is able to bind to the Bcl-2 promoter and activate an anti-apoptotic program (Gupta et al., 2019). In addition, we demonstrated that cell apoptosis is induced by As(III) through activation of the mitochondrial pathway (**Figures 3**, **8**). Thus, we hypothesized that UDCA provides protection against apoptosis induced by As(III) by activating Nrf2 inhibit the mitochondrial pathway. Nrf2 pathway and downstream related proteins are closely related to p53 mediated apoptosis related proteins. According to the STRING database, p53 and nrf2 form a mutual control network, as shown in protein-protein interaction (PPI) in **Figure 9A**. In subsequent experiments, we found As(III) can induce the decrease of Bcl-2 expression, and the increase of Bax expression. Most importantly, As(III) can also activate mitochondrial apoptosis pathway and upregulation of p53 protein levels. Cell death processes shown in this study were further promoted through p53, and C release into the cytosol. Thus, downregulation of proapoptotic proteins, p53 and Bax, reduced Cyto C release in cytosol, and upregulation of the anti-apoptotic proteins, Bcl-2, strongly suggested that the protection of UDCA in As(III)-induced LO2 cells. Herein, we discovered that knockdown of Nrf2 by siRNA-Nrf2 transfection reversed the UDCA-induced elevation of p53 under As(III) exposure and also weakened the influences of UDCA on LO2 cell viability loss and apoptosis. These findings indicated that activating the Nrf2 pathway is implicated in the hepatoprotective activity of UDCA. Similar to our results, previous studies showed that other products, such as oleanolic acid, also alleviated liver injury *via* the activation of Nrf2 signaling (Reisman et al., 2009). Based on findings above, it is reasonable to speculate that Nrf2 can be a common molecular target shared by somes hepatoprotective cholic acid compounds compounds.

## Conclusion

In summary, this present study revealed that UDCA afford therapeutic and prophylactic efficacy against As(III) induced cytotoxicity through its anti-oxidation and anti-apoptosis. Our data support the notion that the protective UDCA trigger antioxidant defense through the Nrf2 and p53 signaling pathway (**Figure 9**). Undoubtedly, it plays a vital role in As(III)-induced hepatotoxicity. These findings confirm our hypothesis that further in-depth studies may establish UDCA bioactive, as a possible candidate for the preventive treatment of arsenic-induced oxidative stress associated liver complications in near future.

## Data Availability Statement

All datasets presented in this study are included in the article/**Supplementary Material**.

## Ethics Statement 

The animal study was reviewed and approved by Institutional Animal Care and Use Committee of Shanghai Institute for Food and Drug Control.

## Author Contributions

CL wrote the paper. CL and LL performed the experiments and prepared the figures. SZ and QH analyzed and commented the results. SJ designed the study and supervised the project. All authors contributed to the article and approved the submitted version.

## Funding

This study was supported by National Key Research and Development Plan of China (No.2017YFC1700800), Shanghai Science and Technology Committee (STCSM) R&D Platform Project (No.18DZ2292200), Shanghai Science and Technology Committee (STCSM) Technical Standard Project (No.18DZ2200900).

## Conflict of Interest

The authors declare that the research was conducted in the absence of any commercial or financial relationships that could be construed as a potential conflict of interest.

## Supplementary Material

Supplementary Figure 1 The expression levels of Nrf2 were silenced by siRNA in LO2 cells. LO2 cells were transfected with Nrf2 siRNA or NC siRNA for 48 h, and then incubated with UDCA. **(A**, **B)** Western blotting analysis of Nuclear or Total Nrf2 in cell lysate. Representative blots were shown. Lamin B and GAPDH were used as loading control. Data are presented as mean ± SD (n = 3). **(A)** Cell viability was evaluated by CCK-8. Data are presented as mean ± SD (n = 6). Significant differences are shown as ***P* < 0.01, compared with the NC siRNA group. ^#^ *P* < 0.05, ^##^ *P* < 0.01, compared with the UDCA group.

## Abbreviations

As(III), Arsenious acid; ALT, alanine aminotransferase; ARE, antioxidant-response element; AST, aspartate aminotransferase; CAT, catalase; DCFH-DA, 2,7-Dichlorodi-hydrofluorescein diacetate; DHE, dihydroethidium; GSH, glutathione; GSH-Px, glutathione peroxidase; HE, hematoxylin & eosin; HO-1; heme oxygenase 1; Keap-1, Kelch-like ECH-associated protein 1; LDH, lactate dehydrogenase; MDA, malondialdehyde; MMP, mitochondrial membrane potential; NQO1; NAD(P)H quinone dehydrogenase 1; Nrf2, nuclear factor E2-related factor 2; PPI, protein-protein interaction; ROS, reactive oxygen species; SDS-PAGE, sodium dodecyl sulfate-polyacrylamide gel electrophoresis; SOD, superoxide dismutase; TSH, total sulfhydryl; UDCA, ursodeoxycholic acid.
