# Supplementary figures and images for: Ursodeoxycholic Acid Protects Against Arsenic Induced Hepatotoxicity by the Nrf2 Signaling Pathway
Source: Front Pharmacol. 2020 Oct 16;11:594496. doi: 10.3389/fphar.2020.594496 (PMC7596389; doi:10.3389/fphar.2020.594496)

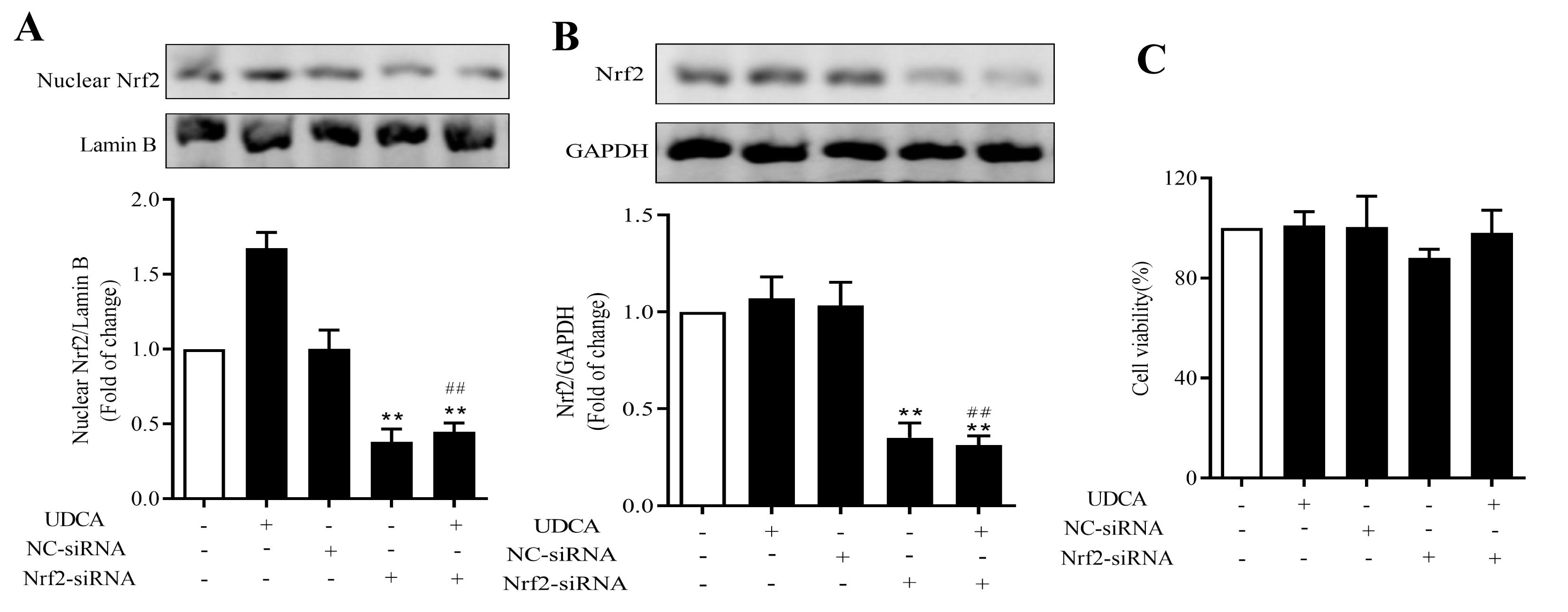

Supplement: Supplementary Figure 1 — The expression levels of Nrf2 were silenced by siRNA in LO2 cells. LO2 cells were transfected with Nrf2 siRNA or NC siRNA for 48 h, and then incubated with UDCA. (A, B) Western blotting analysis of Nuclear or Total Nrf2 in cell lysate. Representative blots were shown. Lamin B and GAPDH were used as loading control. Data are presented as mean ± SD (n = 3). (A) Cell viability was evaluated by CCK-8. Data are presented as mean ± SD (n = 6). Significant differences are shown as **P < 0.01, compared with the NC siRNA group. # P < 0.05, ## P < 0.01, compared with the UDCA group. [file Image_1.tif]
